# Meteorin-Like Protein Levels Decrease in Patients With Acute Ischaemic Stroke

**DOI:** 10.7759/cureus.32042

**Published:** 2022-11-30

**Authors:** Ibrahim Halil Yasak, Mustafa Yilmaz, Mehmet C Goktekin, Ramazan Giden, İsmail Koyuncu

**Affiliations:** 1 Department of Emergency Medicine, Faculty of Medicine, Harran University, Sanlıurfa, TUR; 2 Department of Emergency Medicine, Faculty of Medicine, Firat University, Elaziğ, TUR; 3 Department of Medical Biochemistry, Faculty of Medicine, Harran University, Sanlıurfa, TUR

**Keywords:** oxidative stress index, total oxidant status, total antioxidant capacity, meteorin-like protein, ischaemic stroke

## Abstract

Introduction: Ongoing research aims to investigate blood-based biomarkers and use them in acute ischaemic stroke (AIS) diagnosis and management of patients with AIS.

Purpose: The purpose of the present study was to investigate the meteorin-like protein (Metrnl) levels secreted by adipose tissue in patients with AIS.

Methods: The study groups included healthy controls (n=30) and patients diagnosed with AIS via magnetic resonance imaging (MRI) in the emergency department (n=35) during the one-year period. The basic laboratory values and Metrnl, total antioxidant capacity (TAC), total oxidant status (TOS), and oxidative stress index (OSI) levels of the patients were compared. The Metrnl levels were measured using enzyme-linked immunosorbent assays.

Results: In the present study, the Metrnl (p=0.001) and TAC (p=0.009) levels decreased significantly, whereas the TOS (p<0.001) and OSI (p<0.001) levels increased significantly in the patients with AIS compared to the healthy controls. Furthermore, a cut-off value of ≤1.63% meteorin-like protein rendered the sensitivity and specificity rates of 91.43% and 71.43%, respectively, in the patients with AIS. In addition, there was a significant negative correlation between the decreased meteorin-like protein levels and the infarct diameter in patients with AIS.

Conclusion: In patients with AIS, the meteorin-like protein levels decreased inversely with the infarct diameter, and at the same time, there was an increase in TOS and OSI levels and a decrease in TAC levels.

## Introduction

Acute ischaemic stroke (AIS) accounts for approximately 87% of all stroke cases and is characterized by a sudden loss of blood flow caused by thrombosis or embolism that blocks the cerebral artery that supplies a certain part of the brain [1]. The cerebral metabolism is largely dependent on glucose and oxygen, which ensures the proper functioning of the glycolysis, tricarboxylic acid cycle, and mitochondrial electron transport chain. The brain is highly susceptible to oxidative damage because of its high and specific metabolic activity. Lipid peroxidation is the main mechanism of oxidative damage induced by the reactive oxygen species (ROS). Oxidative stress occurs as a result of the alteration in the balance between ROS production and antioxidant defenses. ROS production increases due to increased oxidative stress in cerebral ischemia, contributing to the resultant ischaemic damage [2]. It was reported that the circulating total oxidant status (TOS) and oxidative stress index (OSI) levels were increased, while the total antioxidant capacity (TAC) levels were generally lower in patients with AIS when compared with the healthy controls [3]. Currently, rapid diagnostic tools for AIS are not available and the stroke is diagnosed upon neurological examination, medical history, and brain neuroimaging. There is ongoing research aimed to investigate blood-based biomarkers and use them in the diagnosis and patient management of AIS [4].

In addition to serving as an energy storage organ, the adipose tissue is an important endocrine organ, and cytokines secreted from this tissue are called adipokines. Meteorin-like protein (Metrnl) is a myokine that regulates energy consumption and inflammation in the adipose tissue. It is mostly expressed in the adipose tissue along with various other tissues, including the muscle, liver, heart, spleen, and central nervous system [5]. In addition, Metrnl is alternatively secreted by the activated macrophages and the macrophages stimulated by the macrophage colony-stimulating factor (M-CSF) [6]. The experimental studies showed that Metrnl antagonized insulin resistance and inhibited inflammation through peroxisome proliferators-activated receptor γ (PPARγ) and metabolic activation [7]. It also stimulates IL-4 expression, increasing the anti-inflammatory cytokines. Metrnl was shown to have been significantly overexpressed in inflammatory skin diseases, including psoriasis and atopic dermatitis, as well as in the synovial membranes of human rheumatoid arthritis [6].

The present study aimed to investigate Metrnl levels in AIS disease.

## Materials and methods

The study was commenced after the prior approval of the local ethics committee (Approval no. 21/01/01). Patients, who presented to the emergency department of the university hospital with a newly occurred neurological deficit during a one-year period (January-December 2021) were included in the prospective study. The basic data of the patients, complaints at presentation, and the basic laboratory test results were recorded in the study data form. The data from the patients diagnosed with ischaemic cerebral infarct (n=35) upon magnetic resonance imaging (MRI) (Magnetom Skyra 3T MR; Siemens Healthcare, Erlangen, Germany) and the control group consisted of 30 consecutive patients who presented to the emergency department with nonspecific symptoms and were considered to have AIS in the differential diagnosis, and whose diffusion MRI was evaluated as normal. In the present study, patients newly presenting with neurological deficits, who were diagnosed with infarct upon MRI, and who consented to participate in the study were included. Patients with a history of cerebral ischaemia or hemorrhage, with intra-cranial mass and any oncological disease, patients who did not undergo MRI, and patients who did not consent to participate in the study were excluded from the study.

Laboratory measurements

Blood samples were collected from all patients at admission. These blood samples were centrifuged for 10 min at 3000 rpm, and the serum was stored at −80°C in aliquots until the day of analysis. White blood cell (3.7-10.1 10e3/μL), hemoglobin (12-18 g/dL), hematocrit (35%-53.7%), and platelet (142-424 10e3/μl) counts were determined with the Alinity HQ (Abbott, Chicago, IL, USA). Serum glucose (70-105 mg/dL), urea (10-50 mg/dL), and creatinine (0.2-1.11 mg/dL) levels were measured by conventional laboratory methods on Atellica Solution (Siemens Healthineers, Germany).

Meteorin-like protein assay

Blood samples were collected from all patients at admission. The samples were then centrifuged for 10 min at 3000 rpm, and the serum was stored at −80°C in aliquots till the day of analysis. The Metrnl levels were measured using enzyme-linked immunosorbent assays (YLA3736HU; Shanghai YL Biotech Co., Ltd., Shanghai, China) according to the manufacturer's instructions. The 96-microplate is pre-coated with human Metrnl antibodies in this kit protocol. Metrnl binds to the antibodies in the 96-plate, which is previously added with Metrnl antibodies. Molecules that are not bound are removed by washing. Then biotinylated human Metrnl antibodies are added to the wells, ensuring that they bind to the meteorite in the sample. Then Streptavidin-HRP is added to bind to the biotinylated Metrnl antibodies. After incubation, the unbound Streptavidin-HRP is removed by the washing step. Then the substrate solution is added, and the color develops in proportion to the amount of human Metrnl. The reaction is terminated by adding an acidic cessation solution and the absorption is measured at 450 nm in the microplate reader (Cytation-1; Biotek, Winooski, VT, USA). Assay range 0.05-15 ng/mL.

Total Antioxidant Level

The TAS level of the samples was measured by the Rel Assay branded commercial kits (LOT: HN20106A; Rel Assay Diagnostics, Gaziantep, Turkey). Trolox, a water-soluble analog of vitamin E, was used as a calibrator. The results were expressed in mmol Trolox equiv./lt.

Total Oxidant Level

The TOS level of the samples was measured by the Rel Assay branded commercial kits (Rel Assay Diagnostics). Hydrogen peroxide was used as a calibrator. The results were expressed in μmol H2O2 equiv./lt.

Oxidative Stress Index

The mmol value in the unit of the TAS test was converted to the μmol unit as in the TOS test during the calculation of OSI, which was expressed as the percentage of the ratio of TOS to TAS levels [8]. The results were expressed as "Arbitrary Unit” and calculated according to the following formula: TOS, μmol H2O2 equiv./lt OSI=TAS, mmol Trolox equiv./lt × 1.

Statistical analysis

The data was statistically analyzed using the Statistical Package for the Social Sciences (SPSS) v.21.0 (IBM Corporation, Armonk, NY, USA) and the MedCalc (Version 10.1.6.0) software package. In the data analysis, the Shapiro-Wilk test was used to test the normality hypothesis for the distribution of the continuous variables. The data without normal distribution were expressed in the median (IQR: interquartile range, where the qualitative data were expressed in percentage values. The Mann-Whitney U test was used to compare two groups and the Chi-Square (cross-tab) was used to compare the categorical data. Spearman’s correlation test was used to investigate the correlation. The receiver operating characteristic (ROC curve) analysis was performed using Metrnl levels with an aim to differentiate the patients with AIS and the controls. The ROC curve analysis results were expressed as % specificity, % sensitivity [area under the ROC curve (AUC), p, 95% confidence interval (CI)]. A P value of <0.05 was considered statistically significant for all the analysis results.

## Results

There was no difference by age (p=0.286) and gender (p=0.534) between the 35 ischaemic patients with AIS and 30 controls included in the present study. Comparing the laboratory results of the study groups revealed the presence of significant inter-group differences in terms of glucose (p<0.001), urea (p=0.002), creatinine (p=0.002), and white blood count (WBC) (p<0.001) levels. However, there was no significant inter-group difference in terms of the hematocrit (HCT), hemoglobin (HGB), and platelet count (PLT) levels. In the present study, the Metrnl (p=0.001) and TAS (p=0.009) levels were significantly decreased, where the TOS (<0.001) and OSI (p<0.001) levels were significantly increased compared to the control group. The basal data of the study groups are given in Table 1.

**Table 1:** Basic data of the patients included in the study *Median (IQR) CAD: Coronary Artery Disease, Metrnl: Meteorin-like protein, TAC: Total Antioxidant Capacity, TOS: Total Oxidant Status, OSI: Oxidative Stress Index

	Control Group	AIS	p
N(F/M)	30 (14/16)	35 (17/18)	0.534
Age*	60.5 (55–72.7)	67 (58–78)	0.286
Glucose* (mg/dL)	97.5 (88–115.25)	129 (106–200)	<0.001
Urea* (mg/dL)	25.68 (19.26 (38.35)	40.66 (30–50)	0.002
Creatinine* (mg/dL)	0.70 (0.50–0.80)	0.80 (0.70–1.20)	0.002
White Blood Count* (10e^3^/μl)	7.35 (6.67–8.23)	9.47 (8.42–15.00)	<0.001
Haematocrit* (%)	43 (38.77–46.32)	41.31 (36.70–43.70)	0.180
Haemoglobin* (g/dL)	13.30 (12.27–15.32)	13.22 (11.81–14.40)	0.406
Platelet* (10e3/μl)	289.50 (222.25–329.25)	265.00 (185.00–316.00)	0.171
Meteorin-like protein (ng/mL)	1.80 (1.65–2.01)	1.56 (1.35–1.59)	<0.001
TAC* (mmol Trolox equiv./lt)	1.36 (1.25–1.64)	1.25 (0.44–1.39)	0.009
TOS* (μmol H_2_O_2_ equiv./lt)	11.35 (10.07–12.56)	16.07 (13.39–18.15)	<0.001
OSI* (Arbitrary Unit)	0.82 (0.62–0.98)	1.25 (1.06–1.45)	<0.001

There was no difference between the Metrnl levels (p=0.170) of the patients with AIS included in the study by gender and between patients with and without a comorbid disease, hypertension (p=0.593), type 2 diabetes mellitus (p=0.193), coronary artery disease (p=0.443). 

**Figure 1:**
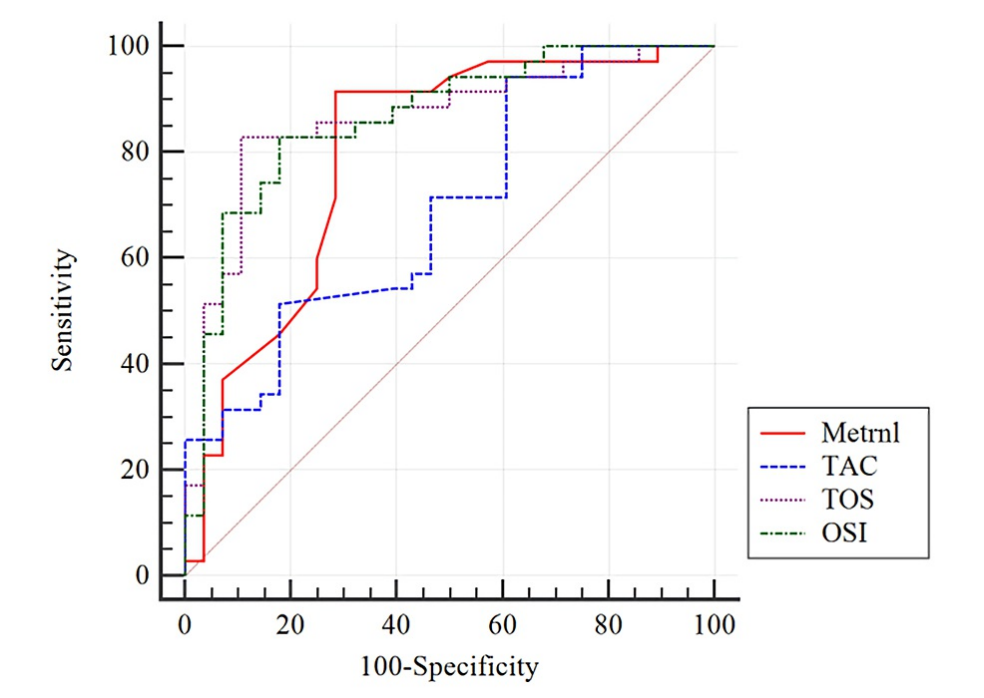
ROC analysis graph for the use of Metrnl, TAC, TOS and OSI differentiation control group and AIS ROC: Receiver Operating Characteristic, Metrnl: Meteorin-like protein, TAC: Total Antioxidant Capacity, TOS: Total Oxidant Status, OSI: Oxidative Stress Index, AIS: Acute Ischaemic Stroke

The ROC analysis performed for differentiating the AIS and control groups found that Metrnl had a sensitivity of 91.43% and a specificity of 71.43% (Figure 1, Table 2).

**Table 2:** ROC analysis results for the use of Metrnl, TAC, TOS and OSI differentiation control group and AIS ROC: Receiver Operating Characteristic, AUC: Area under the Receiver Operating Characteristic curve, CI: Confidence interval, Metrnl: Meteorin-like protein, TAC: Total Antioxidant Capacity, TOS: Total Oxidant Status, OSI: Oxidative Stress Index, AIS: Acute Ischaemic Stroke

	Cut-off	AUC	Youden index J	95% CI	p	Sensitivity	Specificity
Metrnl	≤1.63	0.790	0.6286	0.677–0.888	<0.001	91.43	71.43
TAC	≤1.46	0.693	0.3357	0.564–0.803	0.004	94.29	39.29
TOS	>12.94	0.861	0.7214	0.751–0.935	<0.001	82.86	89.29
OSI	>1.01	0.863	0.6500	0.753–0.937	<0.001	82.86	82.14

There was a significant negative correlation between the Metrnl levels and ischaemic area diameter (r=−0.297, p<0.018) in patients with AIS.

## Discussion

The present study mainly aimed to investigate the Metrnl levels at admission to the emergency room in patients with AIS, and found that the Metrnl and TAC levels were significantly decreased in patients with AIS compared to the control group, where the TOS and OSI levels significantly increased. The present study also found a negative correlation between the diameter of the infarct and the Metrnl levels.

The cerebral metabolism is largely dependent on glucose and oxygen, which ensures the proper functioning of the glycolysis, tricarboxylic acid cycle, and mitochondrial electron transport chain. The brain is highly susceptible to oxidative damage due to its high and specific metabolic activity [2]. The decreased ATP levels induced by hypoxia during stroke trigger oxidative damage, leading to neuronal damage. Lipid peroxidation is the main mechanism of oxidative damage induced by ROS [9]. The ROS imbalance results in apoptosis, blood-brain barrier deterioration, inflammation, edema formation, autophagy, and other pathophysiological events in the AIS. Relevant studies suggested that along with neurons other cells played a role in the pathogenesis of ischemia. Microglia and astrocytes are activated within hours following AIS, leading to the secretion of cytokines and chemokines those results in the infiltration of leukocytes [10]. The oxidative stress especially induced in cerebral ischemia by inflammation and re-perfusion increases ROS production, which may cause direct tissue damage through various mechanisms in the central nervous system [11]. Even though rapid re-oxygenation through reperfusion is a desirable condition in ischaemic stroke to reduce metabolic stress, re-oxygenation per se can also contribute to the occurrence of reactive oxidants [12]. A study by İçme et al. reported increased levels of TOS and OSI in both ischaemic and hemorrhagic stroke [13]. A study by Ghonimi et al. which aimed to investigate the TAC levels in patients with AIS (n=60) and healthy controls (n=30) with similar age and gender characteristics, found that patients with AIS had significantly lower TAC levels compared to the controls [14]. In the present study, the fact that there was a significant decrease in the TAS level along with a significant increase in the TOS and OSI levels was consistent with the results reported in the literature.

The growing evidence that adipokines played a role in atherosclerotic and vascular reshaping processes due to their effects on endothelial and flat muscle cells indicated that the said molecules may serve as promising biomarkers for cardiovascular and cerebrovascular diseases [15-17]. Adipokines may directly or indirectly affect diseases thanks to their atherosclerotic, vascular, inflammatory, or anti-inflammatory effects. Metrnl was shown to have improved glucose tolerance and stimulated an eosinophil-related increase in interleukin (IL)-4 expression [18]. Furthermore, a number of studies confirmed the association between Metrnl and diabetes mellitus and reported that serum Metrnl levels were lower in patients with type 2 diabetes mellitus compared to the healthy controls [19]. A study by Bridgewood et al. on microvascular diseases found that the serum Metrnl concentrations were inversely proportional to kidney function and diabetic nephropathy [20]. The overexpression of Metrnl increases the expression of anti-inflammatory genes, including IL-10 and transforming growth factor (TGF)-b, but it was reported to have reduced the expression of pro-inflammatory genes, including tumor necrosis factor alpha (TNF-α), interferon (IFN)-c and IL-1b [18]. IL-1β, TNF-α, and IL-6 are the three main pro-inflammatory cytokines that trigger and exacerbate the inflammatory response following a stroke [21]. The increased inflammatory effect in AIS may also be caused by the increased inflammatory processes due to reduced Metrnl levels. Previous studies reported that IL-1, which increased after ischaemic stroke or other risk factors, targeted astrocytes and increased the expression of IL-6, TNF-a, MMP-9, and chemokines [22]. Decreased Metrnl levels may lead to an increased expression of pro-inflammatory genes, including TNF-a, IFN-c, and IL-1b. Zhang et al. reported in their experimental study that IL-4 supported oligodendrocyte regeneration and re-myelination, and intranasal administration of IL-4 nanoparticles following a stroke improved the white matter integrity in wild mice and reduced the long-term sensorimotor and cognitive deficiencies [23]. Metrnl was shown to have induced an increase in the IL-4 expression of interleukin-4 [18]. Decreased Metrnl might have led to a decrease in Il-4 expression, therefore, resulting in a weakening in the maintenance of white matter integrity. IL-10, an important anti-inflammatory cytokine with inhibitory effects on various immune cells, was reported to have played an immunomodulatory role in improving neuroinflammation [22]. It was reported that IL-10 had generally no significant relation with the risk of ischaemic stroke, but was associated with macro-vascular and micro-vascular disease [24]. Decreased Metrnl may also lead to a decrease in IL-10 levels, delaying neuroinflammatory improvement.

This study has some limitations. First, this study was presented as a preliminary study, the onset time of patient symptoms, the time between blood samples, and the verbal expression of patient relatives could not be determined because it was not clear. The National Institutes of Health Stroke Scale (NIHSS) score could not be calculated due to patient-related reasons.

## Conclusions

Metrnl is a myokine that regulates energy consumption and inflammation in the adipose tissue. It is mostly expressed in the adipose tissue along with various other tissues, including the muscle, liver, heart, spleen, and central nervous system. In addition, Metrnl is alternatively secreted by the activated macrophages and the macrophages stimulated by the M-CSF. The Metrnl levels were lower in patients with AIS and further decreased as the infarct diameter increased. This suggests at the same time that the increased TOS and OSI levels and decreased TAC levels might also play a role in the decrease in the Metrnl levels.
